# Interventions to improve self-management of adults living with HIV on Antiretroviral Therapy: A systematic review

**DOI:** 10.1371/journal.pone.0232709

**Published:** 2020-05-11

**Authors:** Habtamu Abera Areri, Amy Marshall, Gillian Harvey

**Affiliations:** 1 Adelaide Nursing School, Faculty of Health and Medical Sciences, the University of Adelaide, Adelaide, South Australia, Australia; 2 School of Nursing and Midwifery, College of Health Sciences, Addis Ababa University, Addis Ababa, Ethiopia; Brown University, UNITED STATES

## Abstract

**Introduction:**

Since its initial recognition, HIV has been responsible for around 35 million deaths globally. The introduction of Antiretroviral Therapy has helped to reduce mortality from HIV. However, the resulting increased longevity has influenced the experience of people living with HIV, which now manifests as a chronic condition requiring effective self-management. This review aimed to identify and evaluate the effectiveness of interventions to improve self-management of adults living with HIV on Antiretroviral therapy.

**Methods:**

The review included published experimental studies addressing interventions to improve self-management of adults living with HIV on Antiretroviral Therapy. Studies were included if they addressed two or more outcomes of self-management, as defined by the Theory of Individual and Family Self-Management. The search covered four databases and was limited to papers published in the English language from 2001 to March 30, 2019. The reference lists of included studies were further searched for additional studies. Two independent reviewers using the Joanna Briggs Institute Meta-Analysis of Statistics Assessment and Review Instrument (JBI SUMARI) assessed the methodological quality of the reviewed papers. Data extraction was undertaken using the JBI SUMARI standardized data extraction tool. As the included papers were not homogeneous, it was not possible to conduct a meta-analysis. A narrative synthesis was undertaken to synthesize the findings of the included studies.

**Results:**

The search identified 337 articles from which 10 experimental and 2 quasi-experimental studies were included. The total participant sample in the included studies was 1661 adults living with HIV. The overall evidence quality of the findings was considered moderate. Many of the studies included in this review comprised multi-component interventions to improve self-management. Skills training, in conjunction with other forms of interventions, particularly phone counseling, was commonly employed and generally effective in improving self-management outcomes. Counseling with a symptom management manual was another employed and effective intervention, followed by technology-assisted self-management interventions. The most common outcomes measured were maintaining medication adherence and quality of life, followed by symptom management, self-efficacy, coping, and social support.

**Conclusions:**

Interventions to improve self-management varied across studies. However, promising outcomes achieved in the majority of studies through interventions comprising a combination of skills training, phone counseling, counseling with symptom management manuals, and technology-assisted interventions.

## Introduction

HIV/AIDS has been responsible for an estimated 35 million deaths from a population of around 78 million people infected with the virus since the start of the global epidemic [1].

The advancement of care and treatment of HIV has significantly improved the life expectancy of adults living with HIV. The introduction of Antiretroviral Therapy (ART) has helped to reduce the death rate and increase longevity for adults with HIV. In turn, this has resulted in the re-labeling of HIV as a chronic condition [2]. In common with other chronic conditions, effective self-management is critically important due to the improved survival of adults living with HIV and on ART [3]. Self-management is the ability of adults living with HIV to manage the physical, psycho-social, and behavioral changes associated with the condition [4].

Grady and Gough [5] noted that self-management is regarded as an essential aspect of managing chronic disease, with a focus on illness prevention and promoting wellness. Adults living with HIV (ALWHIV) are required to self-manage their course of treatment, including physical health practice, psychosocial functioning, and daily adjustment to living with chronic illness [2, 5]. The need for promoting self-management behaviors is increasing in HIV treatment [6] as improved self-management can help to reduce the symptoms of the disease and enable decision-making regarding the disclosure of HIV status [7]. Self-management also contributes to improved health status by building the individual’s knowledge, skills, and confidence to manage their illness, including the prescribed treatment schedule [8]. Overall, improved self-management could help to prevent the worsening of HIV, reduce hospital visits, improve clinical care and outcomes, and reduce the burden of comorbidities [8, 9]. Skills that have been identified as necessary for effective self-management include maintaining medication adherence, managing negative emotions, adapting to the nature of the illness, problem-solving, using the available resources, coping with HIV-related conditions, and developing positive social and family relationships [2, 10, 11]. Identifying interventions to improve self-management is critical for implementing and maximizing treatment benefits for ALWHIV on ART.

**Fig 1:**
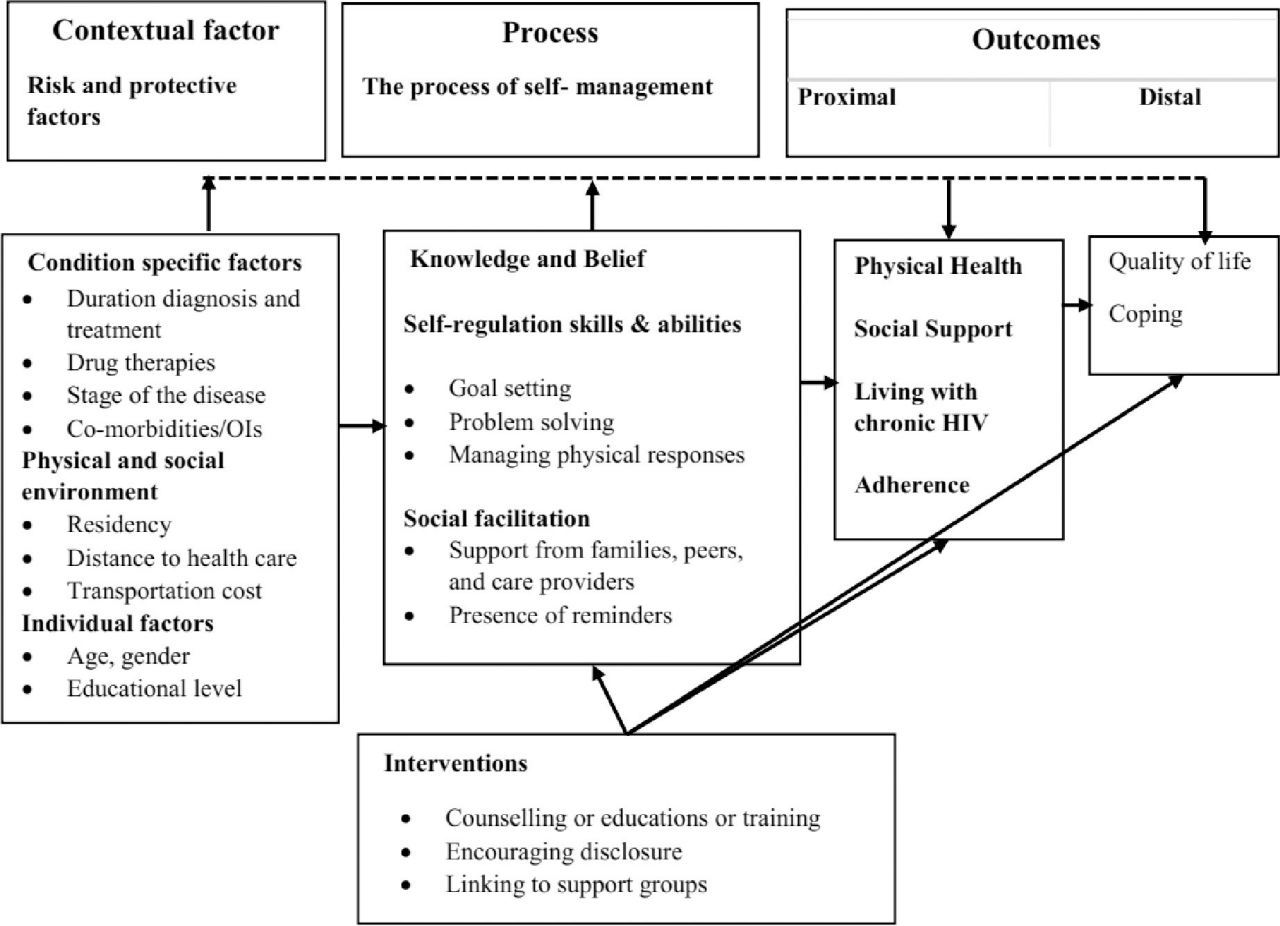
Individual and Family self-management Theory[17].

The review reported here was guided (Fig 1) by the Individual and Family Self-Management Theory (IFSMT). The theory represents the complex, multi-dimensional nature of self-management and encompasses context, process, and outcome components. Contextual factors include condition-specific considerations, the physical and social environment, and individual and family factors. The self-management process–the typical target for self-management interventions–includes factors relating to knowledge and beliefs, self-regulation skills and abilities, and social facilitation. Outcomes are classified as proximal or distal and encompass physical health, psychological and behavioral health outcomes [12, 13]. Concerning HIV, proximal self-management outcomes related to physical health practice, social support, and self-management, living with a chronic HIV condition, and maintaining medication adherence [2, 3, 11, 14–16]. Distal health outcomes are focused on the quality of life and coping with HIV-related conditions [2, 16, 17]. This theoretical classification of the intervention processes and outcomes of self-management was applied to frame the conduct of the systematic review.

Whilst there is increasing awareness of and attention to interventions to improve the self-management practice of ALWHIV, there have been few attempts to summarize the existing evidence base. A systematic review focused on the specific intervention of a self-management education program for people living with HIV for five or more years and found a statistically significant improvement in physical, psychological, knowledge, and behavioral outcomes [13]. Another review conducted on general chronic disease self-management interventions in sub-Saharan Africa and inferred relevance to the HIV population in the region, although concluded that there was limited and inconclusive evidence to inform policy and practice in the area[18]. To date, there have been no systematic reviews in a comprehensive way that focused on the effectiveness of the overall self-management interventions for ALWHIV. This is the aim of the current review, namely, to identify and evaluate the effectiveness of interventions that have been employed to improve the self-management of adults living with HIV on ART.

## Methods

### Protocol registration

The systematic review was conducted following the Joanna Briggs Institute methodology for systematic reviews of the effectiveness of evidence (Joanna Briggs Institute 2014). The protocol was registered on PROSPERO, the international prospective register of systematic reviews (registration number CRD42018118257), and was conducted according to the prior registered protocol [19].

### Search strategy

The search strategy was developed in conjunction with a University Librarian and applied the PICO format to specify the population of interest and intervention. The search strategy applied a three-step process to identify published papers. The first step of the search was limited to PubMed and CINAHL to identify keywords and terms contained in the title and abstract, and the index terms used to describe articles. The second step search was conducted using the pre-identified keywords and index words across four databases: PubMed, Google Scholar, CINAHL, and EMBASE. Initial keywords were: “Interventions”, “self-management”, "self-care", "Strategies", “Improve”, “ART Drugs”, “ARV drugs”, “HAART”, “HIV patients”, “interventions to improve self-management among ART patients”. The search terms were used separately and in combination using Boolean operators “OR” or “AND”. Finally, for the third step, the reference lists of all identified reports and articles were searched for additional studies (S1 Appendix).

## Types of studies

The review considered only peer-reviewed published papers of both experimental and quasi-experimental studies, including randomized controlled trials (RCTs) and non-randomized controlled trials on interventions to improve the self-management of ALWHIV on ART. The reason for including only published experimental studies was to get the most reliable possible evidence on self-management interventions to inform recommendations about which interventions are the most efficacious and should be implemented.

### Population

The review included studies on adults living with HIV on ART (limited to adults as most studies used age (18 +) as an inclusion criterion) and published in the English language from January 2001 to March 30, 2019. The start date of 2001 was selected as it reflected the time from when the main comprehensive research on HIV self-management commenced. The review considered participants receiving HIV care and treatment in health care facilities, community settings, and nursing homes. Finally, studies that measured at least two outcomes of self-management, as defined by the Individual and Family Self-Management Theory [17], were included in this review. According to the theory, self-management is a holistic behavioral practice that typically requires multiple interventions to achieve improvements in multi-dimensional outcomes [11, 17]. Therefore, out of the primary outcome categories specified in the Individual and Family Self-Management Theory, the review planned to include studies that targeted at least two outcomes as a single outcome measure was deemed insufficient as an indicator of effective self-management. The review excluded studies with children as participants as they may be unable to exercise independent self-management. Additionally, grey literature was excluded, as well as studies that reported only a single outcome, or unrelated outcome measures. Pilot studies and studies with a sample size of less than 40 [20] were also excluded.

## Intervention(s)

The review considered studies that evaluated interventions intended to improve the self-management of ALWHIV on ART. These are defined as interventions that help ALWHIV to manage their condition and achieve optimal functioning actively. Interventions could address one or more of the domains in the IFSMT and included training or education about self-management, provision of social support, counseling on HIV treatment, peer mentoring, provision of technology-assisted supports, such as text messaging, phone calls/counseling and online support.

## Comparator(s)

The review considered studies that compared self-management interventions to usual care or standard care.

## Outcomes

As described, the review included studies that measured two or more self-management outcomes. The protocol [19] distinguished between proximal [primary] and distal [secondary] outcomes, as follows. Proximal [Primary] outcomes included immediate self-management behaviors such as maintaining medication adherence, mobilization of social support, living with chronic HIV conditions, physical health practice, coping, and self-efficacy. Distal [secondary] outcomes were concerned with the long-term effects of self-management interventions and included quality of life, reduced mortality, morbidity, and hospital visits. Deviation from the proposed protocol encountered related to some of the anticipated outcomes as these were addressed differently in different studies. As a result, the review focused more generally on outcomes, without explicitly distinguishing between proximal and distal outcomes.

### Study selection

Following the search, all identified citations were collated and uploaded into EndNote version 8.0, and duplicates were removed. Titles and abstracts were then screened by two independent reviewers (HA & AM) for assessment against the inclusion criteria for the review. Potentially relevant studies were retrieved in full, and their citation details imported into the Joanna Briggs Institute System for the Unified Management, Assessment, and Review of Information [21]. The full text of selected citations was assessed in detail against the inclusion criteria by two independent reviewers (HA & GH). Reasons for exclusion of full-text studies that did not meet the inclusion criteria were recorded and reported in the systematic review. Any disagreements that arose between the reviewers at each stage of the study selection process were resolved through discussion.

### Assessment of methodological quality

Eligible studies were critically appraised by two independent reviewers (HA & GH) using the Joanna Briggs Institute Meta-Analysis of Statistics Assessment and Review Instrument [21] for experimental and quasi-experimental studies. Any disagreements that arose were resolved through discussion. The results of critical appraisal were reported in narrative form and a table. A minimum of 50 percent “yes” ratings on the JBI critical appraisal instrument for RCT and quasi-experimental studies were applied as a cut-off point for inclusion in the review [22]. The level of evidence of the findings was also assessed using GRADE pro software.

### Data extraction

Data extraction was undertaken by two independent reviewers (HA & AM). One reviewer (HA) initially extracted data from the included papers using the JBI-SUMARI standardized data extraction tool, and a second reviewer (AM) checked the extraction. Interpretation and data extraction differences resolved through discussion. The data extracted included specific details about the participants, study methods, sample size, study design, publication year, completion rate, study area, interventions, and outcomes of significance to the review objective.

### Data synthesis

Differences in populations, interventions, comparators, and outcomes of the included studies did not allow for direct comparison, and therefore statistical pooling of data was not possible for this review due to significant heterogeneity across studies. Consequently, the results synthesized in narrative and tabular form to aid data presentation.

## Results

### Selection of the studies

**Fig 2:**
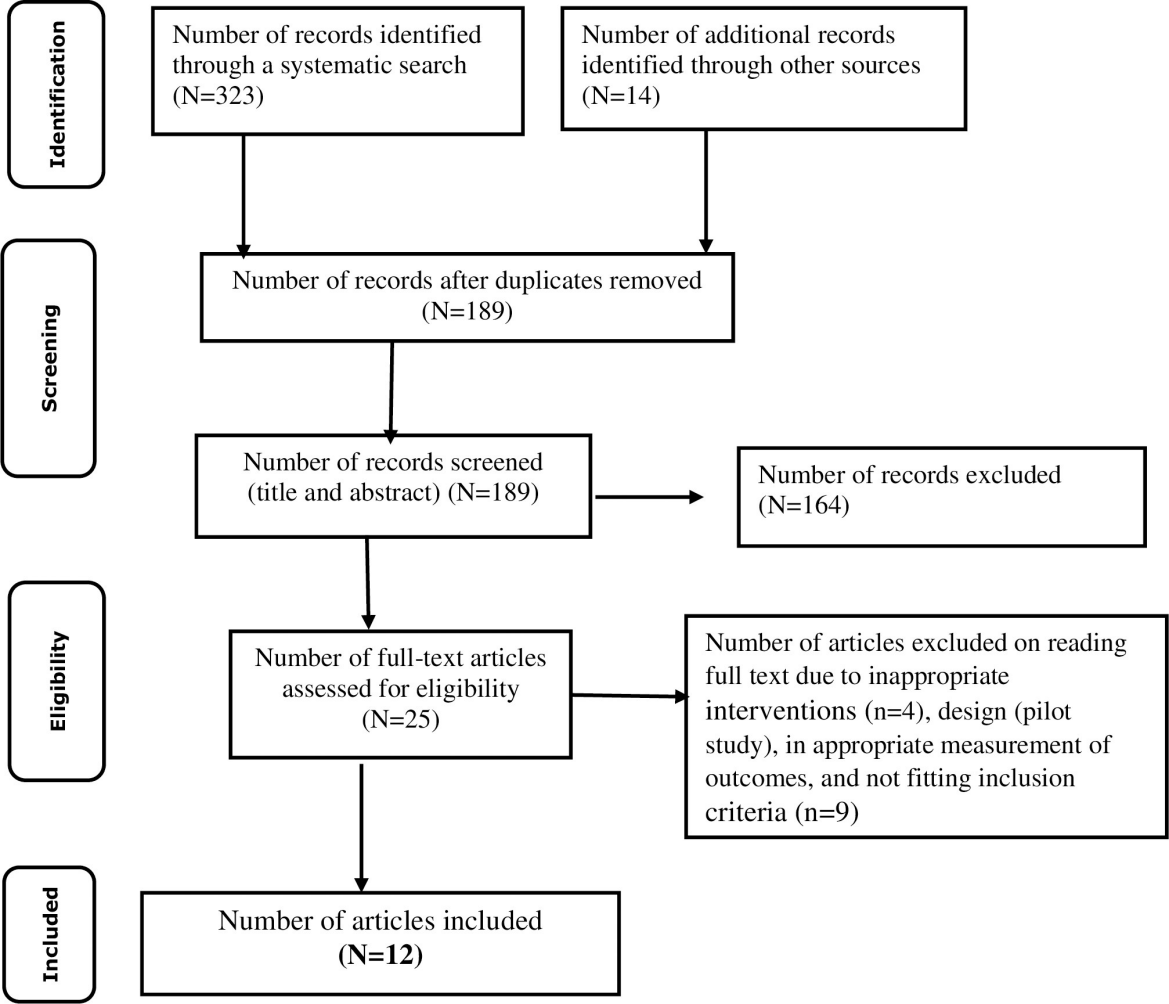
Flowchart of the study selection and inclusion process [23].

Initial database searching recovered 337 unique records, which were catalogued in citation management software (EndNote X8). Following the removal of duplicate studies, the titles and abstracts were evaluated, and studies were excluded if they did not meet the pre-specified inclusion criteria. Then, 189 studies were included for further assessment. After reviewing the full text, studies that were not about HIV self-management had a sample size of < 40 participants, included only medication adherence as an outcome, and pilot studies were excluded, leaving 25 studies. A further four studies were excluded based on the interventions that were evaluated as they did not fit with the objectives of the review. Nine studies were also excluded on the grounds of eligibility criteria—detailed information in Fig 2 [23].

### Methodological quality

The appraisal results for the included studies are outlined in Tables 1 and 2. The review included 10 RCT and 2 quasi-experimental studies, and therefore, according to the JBI systematic review guideline, the level of evidence is ranked as level 1b [21]. Using the GRADE pro software, the level of evidence of the review findings rated as moderate (S1 Table). All studies included in the review got over 50% “yes” answers in the critical appraisal checklist. Two of the studies (quasi-experimental) lacked true randomization [24, 25], while in two other studies, the process of randomization was not clearly described [26, 27]. Concealment of group allocation occurred in one study [28], was unclear in three articles [26, 29, 30], and did not occur in the remaining studies. In many of the included studies, blinding did not occur due to practical difficulties. The study reported by Kalichman et al. [28] was the exception as it did employ blinding procedures. All studies included in the review reported in a reliable way, the outcome measured.

**Table 1:** Methodological quality of quasi-experimental study.

Citation	Q1	Q2	Q3	Q4	Q5	Q6	Q7	Q8	Q9
Côté et al. 2015 [24]	Y	Y	Y	Y	Y	Y	Y	Y	Y
Chiou et al. 2006 [25]	Y	U	Y	Y	Y	Y	Y	Y	Y
%	100	50	100	100	100	100	100	100	100

JBI Methodological quality appraisal Checklist to be score as “Yes, No or Uncertain”.Q1. Is it clear in the study what is the “cause” and what is the ‘effect’ (i.e. there is no confusion about which variable comes first)? Q2. Were the participants included in any comparisons similar? Q3. Were the participants included in any comparisons receiving similar treatment/care, other than the exposure or intervention of interest? Q4. Was there a control group? Q5. Were there multiple measurements of the outcome both pre and post the intervention/exposure? Q6. Was follow up complete and if not, were differences between groups in terms of their follow up adequately described and analyzed? Q7. Were the outcomes of participants included in any comparisons measured in the same way? Q8. Were outcomes measured in a reliable way? Q9. Was an appropriate statistical analysis used?

**Table 2:** Methodological quality of Randomized controlled trial.

Citation	Q1	Q2	Q3	Q4	Q5	Q6	Q7	Q8	Q9	Q10	Q11	Q12	Q13
Kalichman et al. 2011 [28]	Y	Y	Y	Y	Y	U	Y	Y	Y	Y	Y	Y	Y
Miles et al. 2003 [29]	Y	U	Y	U	N	Y	Y	Y	Y	Y	Y	Y	U
Smith et al. 2003 [30]	Y	U	Y	N	N	N	Y	Y	Y	Y	Y	Y	Y
Webel AR. 2010[26]	U	U	Y	N	U	U	N	Y	Y	Y	Y	Y	Y
Giordano et al. 2016 [31]	Y	N	Y	N	N	Y	N	Y	Y	Y	Y	Y	Y
Inouye et al. 2001[27]	U	N	Y	N	N	N	Y	Y	Y	Y	Y	Y	Y
Johnson et al. 2010 [32]	Y	N	Y	N	N	N	Y	Y	Y	Y	Y	Y	Y
Millard et al. 2016[33]	Y	N	Y	N	N	U	Y	Y	Y	Y	Y	Y	Y
Chiou et al. 2004 [34]	Y	N	Y	N	U	N	Y	Y	Y	Y	Y	Y	Y
Webel et al. 2018 [35]	Y	N	Y	N	N	N	Y	Y	Y	Y	Y	Y	Y
%	80	10	100	10	10	20	80	100	100	100	100	100	90

JBI Methodological quality appraisal Checklist to be score as “Yes, No or Uncertain”. Q1. Was true randomization used for the assignment of participants to treatment groups? Q2. Was allocation to groups concealed? Q3. Were treatment groups similar at the baseline? Q4. Were participants blind to treatment assignment? Q5. Were those delivering treatment blind to treatment assignments? Q6. Were outcomes assessors blind to treatment assignment? Q7. Were treatment groups treated identically other than the intervention of interest? Q8. Was follow up complete, and if not, were differences between groups in terms of their follow up adequately described and analyzed? Q9. Were participants analyzed in the groups to which they were randomized? Q10. Were outcomes measured in the same way for treatment groups? Q11. Were outcomes measured in a reliable way? Q12. Was an appropriate statistical analysis used? Q13. Was the trial design appropriate for the topic, and any deviations from the standard RCT design accounted for in the conduct and analysis?

### Characteristics of included studies

Detailed information about the setting, participants, design, interventions, outcomes, and summary of the results is provided in Table 3. The studies included in the review were undertaken in four different countries: eight were from the USA [26–32, 35], two from Taiwan [25, 34], one from Canada [24], and one from Australia [33]. None of the included intervention studies were undertaken in African countries or other middle- and low-income countries.

**Table 3:** Characteristics of included studies—quasi-experimental study.

Authors	Country and setting/context	Participant characteristics	Design and groups	The main description of results
Côté, et al.,[24]	**Canada**	A total of 179 participants recruited from age group 18 years and older and on ART for at least 6months.	**Quasi-experimental**	The analysis used an intention-to-treat approach and the completion rate of the intervention was 74% (73/99). The mean age of the participants was 48 years with SD of 8.4 and a range of 23–73 years. The mean duration of HIV diagnosis and treatment was 14 and 11 years respectively. Adherence was improved in the intervention group compared to the traditional follow-up. The interventions improved symptom-related discomfort (P<0.05) and mobilization of social support (p<0.05). The intervention group had a better CD4 count compared to the control group (*P* < .05). However, there was no change in self-efficacy.
	**Setting/context** Two University hospitals		**Intervention** (n = 99) Participants received virtual follow up which was technology-assisted teaching focusing on self-assessment skills, reinforcing and motivational skills, managing side effects, emotional management/coping skills, problem-solving process, establishing, maintaining, and strengthening social relation. Around 140 virtual nurse video clips presented. Four intervention sessions carried out over 6 to 8 weeks, and measurement took place at 6 months of follow up.	
			**Control** (n = 80): usual care that covered medication, symptoms, and problems encountered	
Chiou et al. 2006 [25]	**Taiwan**	67 participants with HIV and on ART were recruited based on the inclusion criteria of being diagnosed as HIV-positive and a CD4 count >200/mm3	**Quasi-experimental**	Most of the participants were male (n = 63), 4 were female. The average duration of HIV diagnosis was 27.39 ± 23.38 months. Median differences in adherence, CD4 count, and quality of life in both experimental groups were statistically significantly better than in the control group (p<0.05). The difference in drug adherence between pre- and post-testing was significant (P<0.01) in experimental groups compared to the control group. After the intervention, the quality of life in the experimental group was better than the control group.
	**Setting/context** The outpatient department for infectious diseases of a Taipei medical Centre, and an AIDS social service agency		**Intervention(n = 45)** Symptom management instructions such as self-care of symptoms, skills training, and telephone counseling were applied to two experimental groups. Both individuals and groups were given self-care/self-management instructions and skills training once a week for 60–90 minutes for a consecutive period of three weeks.	
			**Control (n = 22): usual care** Offered comprehensive symptom management instructions.	
Kalichman et al. 2011 [28]	USA	A sample of 40 participants aged 18 years and older were recruited among adults on ART with a self-reported adherence less than 95% in the last month of therapy.	**RCT, blinded**	The approach of analysis was intention-to-treat and the self-management counseling session and the completion rate was 99%. 26 participants were male and 14 were female. The mean age of the participants was 51 (SD, 4.7) years. The self-regulation counseling delivered by phone call demonstrated significant improvements in adherence compared to the control on the four follow up assessment measures (p < 0.01). Gains in adherence were paralleled with increased self-efficacy (p < 0.05). The behavioral self-management counseling condition demonstrated greater self-efficacy for medication adherence at the follow-up (P<0.05). Overall, participants who received behavioral self-management counseling reported a greater number of adherence strategies at the 4 months to follow up than the control condition.
	**Setting/context** Infectious disease clinics in Atlanta, Georgia		**Intervention (n = 21)** Behavioral counseling and cell phone counseling grounded in the self-management model. Contacted by phone called and received a 45-minute counseling session after pill counts that were used as feedback for self-regulation counseling twice a week. The intervention was provided for the four-month duration, and a measurement took place after 4 months.	
			**Control (n = 19):** Usual care, the participants received a phone call for pill count, but the call was not used for feedback on adherence or counseling.	
Miles et al. 2003 [29]	USA	Women (n = 109) were recruited from home to participate in maternal self-care symptom management interventions.	**RCT: Blinded**	The analysis approach was intention-to-treat and the completion rate was 51% (30/59). The mean age of the participants was 37 (SD, 9.5) years. Findings indicated a statistically significant effect of self-care symptom management interventions on the perception of stigma and physical health function (P<0.05). The mothers in the intervention group had lower stigma scores and higher physical function. For the intervention group, there were improvements in both emotional distress and health outcomes. For the control group, there was a significant decline in physical function and overall role function. Overall, interventions reduced emotional distress especially on HIV stigma and improved general health status including quality of life (P<0.05)
	**Setting/context** Two tertiary care University-based infectious disease clinics in the Southeast Carolina state, with nine recruited from HIV care agencies.		**Intervention (n = 59)** Cognitive reframing training on HIV self-care symptom management and provision of symptom management modules. The follow-up period was for six months. The cognitive reframing training involved teaching, interactive discussion, role-play, and problem-solving, and the intervention was embedded in therapeutic communication.	
			**Control (n = 50):** usual care/ focus on health problems rather than self-care symptom management	
Webel. 2010 [26]	USA	The sample comprised 89 eligible HIV infected adult participants who self-identified as female and spoke fluent English.	RCT	The analysis approach was intention-to-treat. All participants were females with a mean age of 47 years (SD 8.16). The peer-based symptom management intervention did not increase medication adherence or symptom management but did improve some aspects of quality of life (HIV mastery, p<0.01 and disclosure worries, p<0.01).
	**Setting/context** San Francisco Bay Area HIV outpatient clinics, HIV/AIDS specific housing, and HIV/AIDS-related community- based care		**Intervention(n = 43)** HIV-infected women followed over 14 weeks. The intervention tested peer-based, HIV symptom management using the curriculum of the Positive Self-Management Program (PSMP) as the content of the sessions. Seven, 2-hour sessions were delivered in-group behavioral skills training.	
			**Control (n = 46):** usual care	
Smith et al. 2003 [30]	USA	Forty-three participants were recruited through referrals made at the discretion of staff clinicians, based on age (18 years or older) and willingness.	RCT	The completion rate of the trial was 96%. The average age of the participants’ was 37 years. Self-management training with the feedback of performance improved adherence to care. The study found also on-going medication counseling and regular consultations that helped to build confidence (better self-efficacy for self-management) and understanding of treatment plan. The adherence level in experimental groups was higher all the time than the control group (P<0.001).
	**Setting/context** North Carolina University Hospital		**Interventions (n = 22)** Participants in the self-management program received individualized patient education and assistance with medication self-management and skills training by a registered pharmacist or nurse. The medication self-management program consisted of information exchange, skills training, counseling, and mobilization of social support. The intervention group followed for 12 weeks.	
			**Control (n = 21):** Usual care/ medication education	
Johnson et al. 2010 [32]	USA	HIV-infected individuals (n = 249), both male and female aged over 18 years included.	RCT	The completion rate of all five sessions was 88%. Most participants were male (n = 226) and 23 were female. The mean age of participants was 46 years (SD 7.9). The mean duration of HIV diagnosis was 14 years and ART treatment 10 years. The intervention was effective in influencing individuals’ efforts to access information (p<0.01) and social support for coping with HIV treatment side effects (p<0.01). Improved coping skills related to HIV treatment side effects had a protective effect on treatment adherence (P<0.05). Interventions focusing on skills related to ART side-effects management showed a promising effect (p<0.001). Odds of non-adherence decreased by 6% per month for the intervention group (p<0.05). A significant overall difference was observed between control and intervention groups regarding coping with side effects. Overall, the intervention improved mobilization of social support (p<0.001), coping with side effects (p<0.05) and adherence to medication.
	**Setting/context** Community agencies and medical clinics		**Interventions(n = 128)** Coping skills for self-management of ART side effects, adherence, and social support. A 60-minute individual counseling session and five problem-solving skills training sessions were conducted.	
			**Control(n = 121):** Usual care/control condition received no active psycho-social interventions	
Inouye et al. 2001 [27]	USA, New York	40 men and women with a diagnosis of HIV participants were recruited, aged 18 years and over.	RCT	Self-management training improved coping. Psychosocial Health showed a significant improvement in mood and coping strategies (P<0.05) and the training significantly improved participants’ mood (p<0.05). The intervention group demonstrated better self-management (p<0.05). However, no significant change was observed in the CD4 count.
	**Setting/context** Participants were recruited through advertisements in newspapers, private physicians, hospital flyer, and AIDS organizations		**Intervention (n = 20)** Received a 7-week individual self-management and coping skills training, which included a 14-session individual intervention for 60–90 minutes on skills training, and problem-solving.	
			**Control (n = 20):** usual care/ waitlist who received standard care.	
Millard et al. 2016 [33]	Australia	A total of 132 homosexual men with HIV were eligible for participation if they self-identified, homosexual were 18 years or older, living in Australia, had adequate English to enable participation and had access to a computer with internet.	RCT	The completion rate was 52% (35/68). The study indicated significant improvement in the intervention group on HIV-related quality of life (p< 0.05); social relationships (p<0.05); health-directed activity (p<0.05); skill and technique acquisition (p<0.05) and health service navigation (p<0.01); Positive Outlook Self-Efficacy (p<0.05) and social participation (p <0.01).
	**Setting/context** HIV-positive gay men recruited through advertisements on social media, community organization websites and print media, AIDS council offices, and primary care clinics.		**Intervention (n = 68)** A training session was focusing on managing the emotional impact of HIV, disclosing HIV status to family and friends, maintaining social connectedness, and disclosure to partners. Seven weeks of intervention for 90 minutes per week	
			**Control(n = 64):** Usual care/ primary health and community-based services and supports without any other additional intervention	
Chiou et al. 2004 [34]	Taiwan	Sixty-seven patients with HIV/AIDS who were receiving ART during the study period and in the past four months and had a CD4 count >200/mm^3^.	RCT	The completion rate was 86%. The mean age of the participants was 32.43 years (SD 6.68). 63 participants were male and 4 were female. Median differences in the customized adherence and quality of life in both interventional groups were statistically significantly improved compared to the control group (P<0.05). Drug adherence in the experimental groups was better than in the control group CD4 in the experimental groups (P <0.01) was better than in the control group (p>0.05. Quality of life in both interventional groups was better (p<0.05) than in the control group, as was self-care ability in managing medication side effects (p<0.05). Overall, the educational program with written manuals helped HIV-positive patients in increasing their self-care knowledge.
	**Setting/context** A medical center and a Catholic AIDS support group in Taipei.		**Intervention (n = 45)** Participants randomly assigned to a 60-minute individual (n = 23) or 90- minute group (n = 22) program on side effects self-care education and skills training once per week for 3 weeks; received telephone counseling and side effects self-care education manual. The intervention lasted for three months.
			**Control (n = 22):** Usual care. No intervention applied to the control group other than telephone counseling.
Webel et al. 2018 [35]	USA	179 participants aged 18 years and older, English speaking, and with at least one chronic condition with HIV were recruited from their home and/or clinic or other community sites according to participant preferences and availability.	**Randomized clinical trial**	The analysis approach was intention-to-treat. The completion rate of the intervention was 52/90 (58%). The navigator program showed variable improvement in outcomes over time: self-blame, as a domain of coping, was lower (p<0.02). Understanding of the chronic nature of HIV self-management showed a positive effect (p<0.01). However, the program did not improve the overall quality of life or social support.
	**Setting/context** Participants were recruited from 3 urban clinical sites specializing in the care of HIV patients.		**Intervention(n = 90)** HIV Navigation Program intervention (Positive Self-management program). Comprehensive palliative care programs, particularly focusing on symptom management through home visits by social workers or nurses and phone calls The intervention was followed for 36 months.	
			**Control (n = 89):** Usual care including medication adherence, general well-being, and hospital use	
Giordano et al. 2016 [31]	USA	460 participants aged 18 years and above and able to speak English or Spanish were included in a study.	**Randomized clinical trial**	Intention-to-treat was the approach of analysis and the completion rate was 90%. The study consisted of 305 males and 155 females. Peer mentoring did not increase re-engagement in outpatient care or effect CD4 count (P>0.05). Mentored participants with a service linkage worker visit and length of stay <7 days had a 1.58 relative risk (95% CI, 1.10, 2.27) of improvement at 6 months compared with other participants.
	**Setting/context** Houston’s Harris Health System\		**Intervention (n = 225)** Peer mentoring and encouragement for self-management. The intervention consisted of 2 in-person sessions in hospital and five telephone calls after discharge over 10 weeks.	
			**Control(n = 235): Usual care;** no focus on retention in care or HIV treatment	

The included studies recruited participants from different settings, including hospitals [24, 30, 31], HIV specialized care clinics [25, 26, 34, 35], community agencies and medical clinics [32], and infectious disease clinics [28, 30], and through advertisement using channels such as social media, agency websites and community organizations [27, 33]. Participant sample sizes varied considerably, ranging from 40 [27, 28] to 460 participants [31].

The mean age of participants was reported in seven studies [24, 26, 28, 29, 32, 34, 35] and ranged from 32.43 years [34] to 51 years [28]. Gender was reported in all except two studies [27, 29]. One study only included female participants [26] and another only male participant [33]. Overall, most of the studies included in the review comprised more male than female participants.

The intervention period ranged from three weeks [27, 33] to 36 months [35], while the measurement period of outcomes ranged from eight weeks [33] to 27 weeks [35]. None of the included studies reported continuing the intervention beyond the final measurement point. The completion rate of participants was reported in all except two studies [25, 27]. This ranged from 51% [29] to 99% [28]. Baseline assessment was made for all included participants, and the finding was compared with post-intervention findings. An intention-to-treat approach was reported in half of the included studies [24, 26, 28, 29, 31, 35]; in the remaining studies, this was unclear or not defined.

### Review findings

#### Intervention strategies

The studies included in the review employed varied intervention strategies. Half of the studies involved skills training intervention [24–27, 29, 30, 32, 34]. Others included phone counselling [25, 28, 30, 34], peer-based intervention [26, 31], provision of an HIV symptom management manual [25, 29, 34] and technology-assisted intervention (phone calling, text messaging and computer-based) [24, 25, 28, 34]. Table 4 summarizes the type of interventions applied, mode of delivery, outcomes, and effectiveness of the intervention in the included studies.

**Table 4:** Summary of the main interventions, mode of delivery, outcomes and effectiveness.

Authors	Intervention/s	Mode of delivery	Outcomes	Effectiveness of the intervention and statistical significance
Côté, et al. 2015[24]	Technology-assisted teaching focused on skills and social support was carried out in two university hospitals.	**Online:** 4 sessions each 20–30 minutes long, offered over 8 weeks provided using a virtual nurse who acted as a coach interacting with the user.	Physical health Outcome • Symptom management • CD4 count Psychosocial Health Outcome • Self-efficacy • Social support mobilization Behavioral Health Outcome • Adherence	The interventions improved: • Symptom management (p<0.05) • CD4 count (p<0.05). • Mobilization of social support (p<0.05) • Adherence No difference in self-efficacy
Chiou et al.2006 [25]	Symptom management instructions, skills training and phone counseling	**Face-to-face and phone counseling:** one-on-one and group teaching on symptom management by HIV care professionals provided once a week for 60–90 minutes, followed by 3 weeks of phone calls.	Physical health Outcome • CD4 count Psychosocial Health Outcome • Quality of life Behavioral Health Outcome • Adherence	The interventions improved: • CD4 count in both intervention groups (one-on-one and group teaching) (P<0.05) • Quality of life in both intervention groups (P<0.05). • Adherence in both intervention groups (P<0.01)
Webel. 2010 [26]	Peer-based HIV symptom management and skills training using the Positive Self-management Program (PSMP) as content for the sessions	**Face-to-face and then phone calls:** 7 sessions each of two hours, led by a trained peer using PSMP in the community through face-to-face sessions and phone calls over 14 weeks.	Physical health Outcome • Symptom management Psychosocial Health Outcome • Quality of life Behavioral Health Outcome • Adherence	The intervention improved: • Some components of quality of life, namely, HIV mastery [sense of self-control] (p<0.01) and disclosure worries (p<0.01). No significant differences in medication adherence or symptom management capacity
Inouye et al. 2001 [27]	Individualized self-management and coping skills training	**Face-to-face:** the intervention was run over seven weeks for 60–90 minutes per session. Two trained clinicians delivered a total of 14 sessions.	Physical health Outcome • Symptom management • CD4 count Psychological outcomes • Coping skills	The intervention improved: • Coping and coping strategies (P<0.05). • Coping skills training resulted in better symptom self-management (p<0.05). No significant change was observed in the CD4 count.
Kalichman et al. 2011[28]	Behavioral self-regulation counseling and cell phone counseling grounded in self-management model	**Initial face-to-face, then telephone calls for follow up:** After initial adherence counseling, participants received 45 minutes of phone counseling per session, 2 times per week for four months from an adherence counselor.	Psychological outcomes • Medication adherence self-efficacy Behavioral Health Outcome • Adherence	The intervention improved: • ART adherence (p<0.01). • Medication adherence self-efficacy (p<0.05)
Miles et al. 2003 [29]	Cognitive reframing training and symptom management modules	**Face-to-face with follow-up telephone calls:** The intervention was carried out in the homes of adults living with HIV (six home visits over 3 months by registered nurses), followed by phone calls.	Physical health outcome • Physical health function Psychosocial Health Outcomes • HIV related stigma • Quality of life	The intervention improved: • Physical health function (p<0.01). • HIV related stigma scores (p<0.001). No significant difference in health-related quality-of-life
Smith et al. 2003 [30]	Individualized medication self-management education by registered health care professionals. The interventions consisted of information exchange, skills training, and social support enlistment	**Face-to-face counseling**: 6-month intervention, including three monthly visits for medication consultations and monthly feedback of adherence performance, delivered by trained nurse or pharmacist.	Psychosocial Health Outcome • Self-efficacy Behavioral Health Outcome • Adherence • Self-management adherence	The intervention improved: • Self-management adherence (p<0.05) • Adherence (p<0.005) No significant difference in self-efficacy (P>0.05)
Giordano et al. 2016 [31]	Peer monitoring training interventions.	**Face-to-face training and phone calls after discharge:** Seven volunteer mentors provided two training interventions in hospital, each lasting between 20 and 45 minutes, and then five follow-up phone calls in the 10 weeks after discharge.	Physical health outcome • CD4 count Psychosocial Health Outcome • Quality of life Behavioral Health Outcome • Adherence	The intervention improved: • Mentored participants with a service linkage worker visited had a 1.58 relative risk (95% CI: 1.10, 2.27) improved adherence level and quality of life at 6 months compared with other participants (p<0.05). The interventions found no change; • Re-engagement in HIV care. • CD4 count (P>0.05)
Johnson et al. 2010 [32]	Individualized counseling and skills training	**Face-to-face:** Five counseling and skills training sessions individually delivered for 60 minutes with a focus on HIV treatment side effects and coping skills by the experienced clinician on HIV care over 3 months.	Psychosocial Health Outcome • Coping skills • Social support mobilization Behavioral Health Outcome • Adherence	The intervention improved: • Adherence (p<0.05) • Coping with side effects (p<0.001) • Providing adequate information improved coping with side effects and adhered to medication(p<0.05) • Social support mobilization(p<0.001)
Millard et al. 2016 [33]	Online peer-based programs delivered via a community-based peer support officer.	**Online:** 7-week program delivered for 90 minutes per week in closed groups with 15 participants per group, comprising information modules and weekly peer-facilitated live chats.	Psychosocial Health Outcome • Self-efficacy • Social support mobilization • Quality of life • Coping ability/adjustment	The intervention improved: • HIV-related quality of life (p<0.05) • Social relationships (p<0.05) • Decreased emotional distress or coping (p<0.05) • Health service navigation (p<0.01) • Positive Outlook Self-Efficacy (p<0.05) • Social participation (p<0.01). • Quality of life (p<0.05)
Chiou et al. 2004 [34]	Individualized self-care education/ group skills training, symptom management manual and phone counseling	**Face-to-face education and skill training with phone counseling to support the face-to-face interaction:** 3 sessions of 60 to 90 minutes each on side effects, self-care education/skills training once per week over 3 weeks. Delivered by HIV care professionals and followed by phone counseling.	Physical Health Outcome • Symptom self-care Psychosocial Health Outcome • Knowledge of self-care Behavioral Health Outcome • Adherence	The intervention improved; • Self-care knowledge (p<0.001). • Adherence (P<0.01) • Symptom self-care ability (P<0.05).
Webel et al. 2018 [35]	Individualized navigator program intervention for HIV patients Comprehensive palliative care programs: home visits by social workers or nurses Phone calls	**Home visit and phone calls by nurses or social workers:** Advanced Practice Nurses and Social Workers delivered the intervention and trained volunteers over 3 years, depending on the need of the participant.	Physical health Outcome • Symptom management Psychosocial Health Outcome • Quality of life • Coping ability • Social support mobilization Behavioral Health Outcome • Living with chronic HIV and Self-management	The intervention improved; • Coping (p<0.02). • Symptom management (P<0.01) • Life satisfaction (p<0.01) and friendship (P<0.05) • Understanding of the chronic nature of HIV self-management showed a positive effect (p<0.01). No change in overall quality of life or social support mobilization

#### Intervention outcomes

Outcomes were categorized under physical health, psychosocial and behavioral outcomes [12, 13] in line with the Individual and Family Self-Management Theory [17]. The physical health outcomes included symptom management and CD4 counts. Behavioral outcomes focused on medication adherence and psychosocial outcomes included self-efficacy, quality of life, coping, and social support. The interventions were summarised as per the Template for Intervention Description and Replication (TIDieR) reporting checklist and guide[36].

#### Interventions and outcomes

The studies included in this review consisted of complex, multi-component interventions that made it difficult to link specific interventions to specific outcomes. The complex nature of the problem requires multiple intervention strategies to improve self-management of ALWHIV on ART. Most of the studies consisted of some form of training interventions in conjunction with counseling or other forms of interventions to improve self-management outcomes. Adherence was the most frequently improved self-management outcome followed by symptom management, coping, and quality of life. The least improved self-management outcome was self-efficacy for self-management (Table 5). To provide additional clarity, the findings of the review are summarized under physical, psychosocial, and behavioral outcomes.

**Table 5:** Summary of the main interventions and improved self-management outcomes.

Interventions	Self-management outcome improved
Technology-assisted interventions [24], Individualized symptom self-management and coping skills training[27], Cognitive reframing training and symptom management modules with phone calls [29], Individualized self-care education/ group skill training, symptom management manual and phone counseling [34], Individualized navigator program intervention for HIV patients with phone calls[35]	Symptom management
Symptom management manuals, Skills training and phone counseling [25], Peer-based HIV symptom management program and skill training [26], Peer monitoring training interventions with follow up phone calls [31], Online peer-based programs [33], Individualized self-care education/ group skill training, symptom management manual and phone counseling [34].	Quality of life
Technology-assisted interventions [24], Symptom management manuals, Skills training, and phone counseling [25], Behavioral self-regulation counseling and cell phone counseling grounded in self-management model [28], Individualized medication self-management education [30], Peer monitoring training interventions with follow up phone calls [31], Individualized counseling and skill training [32], Individualized self-care education/ group skill training, symptom management manual and phone counseling [34]	Maintaining Adherence
Technology-assisted interventions [24], Individualized counseling and skill training [32], Online peer-based programs [33]	Social support mobilization
Individualized symptom self-management and coping skills training[27], Cognitive reframing training and symptom management modules with phone calls [29], Individualized counseling and skill training [32], Online peer-based programs [33], Individualized navigator program intervention for HIV patients with phone calls[35].	Coping skills
Behavioral self-regulation counseling and cell phone counseling grounded in self-management model[28], Online peer-based programs[33]	Self-efficacy for self-management

#### Physical health outcomes

Physical health self-management outcomes include symptom management and CD4 count [12]. Changes in physical health outcomes were evaluated in four papers [24, 26, 27, 34] and two of the four studies demonstrated significant improvements in the intervention groups. A quasi-experimental study conducted by Côté et al. [24] found better symptom management among participants who received virtual follow up (when involved in a technology-assisted teaching session with a virtual nurse) in the experimental group compared to the control group (p< 0.05). An RCT study conducted by Webel [26] reported that a peer-based symptom management program did not significantly improve symptom management. Another quasi-experimental study found that symptom management programs increased self-care ability through multiple teaching sessions and providing written manuals for people living with HIV [34]. An RCT conducted by Inouye et al. [27] found no significant difference between the intervention group who received individualized self-management and coping skills training for seven weeks and the control group about CD4 count (p>0.05). However, an RCT study conducted by Chiou et al. [34] evaluating skills training found a statistically significant improvement in CD4 count in the intervention group (p<0.01). Overall, therefore, the findings relating to the impact of self-management interventions on physical health outcomes are inconclusive.

#### Behavioural health outcomes

Maintaining medication adherence is the main behavioral outcome targeted by HIV self-management interventions [12]. Adherence to medication was reported in seven studies [24–26, 28, 31, 32, 34]. A study conducted by Côté et al. [24] found that adherence was significantly improved among participants who received virtual follow up through technology-assisted teaching. Chiou et al. [25] also found a statistically significant (p<0.01) pre/post-test difference amongst participants who received instructions on self-care of symptoms through skills training and phone counseling. An RCT conducted by Kalichman et al. [28] demonstrated that phone counseling intervention significantly improved adherence (p<0.01). In contrast, Webel [26] found that a peer-supported symptom management intervention did not increase adherence. Chiou et al. [34] found a statistically significant difference in adherence amongst an experimental group who received self-care training. Another RCT study conducted by Johnson et al. [32] also found that an intervention focusing on skills of ART side- effect management significantly improved medication self-management (p<0.05). In summary, interventions to improve medication adherence appear to be generally effective, although less so when delivered via peer mentoring.

#### Psychosocial outcomes

Psychosocial outcomes (quality of life, social support, self-efficacy, and coping) were reported in many of the papers. Quality of life was reported in eight studies [25–27, 29, 31, 33–35]. Of these, Chiou et al. [34] found a statistically significant improvement in the quality of life among the intervention group who received education about side effects and skills training once per week for 3 weeks and a 3-month symptom management intervention (P<0.05). An RCT conducted by Millard et al. [33] also indicated a significant improvement with regards to the quality of life amongst the experimental group who received self-management education (p<0.05).

Similarly, Miles et al. [29] reported that symptom management training interventions improved the quality of life. Chiou et al. [25] also found the quality of life improved in an experimental group provided with symptom management support, skills training and phone counseling (P< 0.05). However, Webel [26] found no improvement in the quality of life amongst participants who received a peer-based self-management intervention. Overall, the interventions studied appear to have a positive impact on the quality of life with the exception of those delivered via peers.

Three studies included interventions to improve the mobilization of social support as part of self-management [24, 33, 35]. A quasi-experimental study conducted by Côté et al. [24] found that social support mobilization was improved among an experimental group that received technology-assisted teaching (virtual follow up) on coping and establishing, maintaining, and strengthening social relationships (p<0.05). Millard et al. [33] conducted an RCT that involved providing an information module on social connectedness, emotional management, and the need for disclosure and reported a significant improvement in social relationships (p<0.01) among the intervention group. However, an RCT conducted by Webel et al. [35] using a Positive Self-Management Program reported no improvement in social support mobilization amongst the experimental group.

Four of the included studies reported that there were changes in self-efficacy for self-management [24, 28, 30, 33]. In one study [24], technology-assisted intervention to improve self-efficacy for symptom management was not significant (p> 0.05). However, in a different study, Kalichman et al. [28] reported that a phone counseling intervention did significantly improve self-efficacy for self-management (p<0.05). Similarly, Smith et al. [30] concluded that ongoing phone counseling and regular consultation improved confidence/self-efficacy for self-management, while Millard et al. [33] also demonstrated that self-management education significantly improved self-efficacy in the intervention groups (p<0.05). These findings suggest that self-efficacy for self-management improvement depends on continuous counseling and education.

Coping with HIV conditions was reported in four studies [27, 32, 33, 35]. A seven-week individual self-management coping skills training intervention found significant improvement in coping and coping strategies (p<0.05) in the experimental group [27]. Johnson et al. [32], also reported that providing information on self-management improved coping with side effects(p<0.001). An online peer-based intervention program demonstrated decreased emotional distress after eight weeks of intervention (p<0.05) [33], whilst an RCT conducted by Webel et al. [35] found that understanding the nature of HIV self-management showed a positive effect on accepting and living with a chronic HIV condition (p<0.01)and lowered self-blame among the intervention group (p<0.05). Therefore, it is possible to conclude that interventions to improve coping were generally useful.

## Discussion

This systematic review narratively synthesized evidence on interventions to improve self-management outcomes, including quality of life, self-efficacy, coping, symptom management, maintaining medication adherence, and mobilizing social support. However, the interventions studied are complex, often multi-component, and highly variable in terms of intensity, duration, and mode of delivery. This heterogeneity makes it difficult to attribute specific outcomes to specific interventions as factors related to intervention design, methods of implementing, and the context in which implementation takes place could all influence the study outcomes. This has implications for future interventional studies, which could be strengthened by including an embedded process evaluation to provide a more comprehensive understanding of what works, for whom, in what circumstances, how, and why[36].

From the evidence to date, it appears that skills training delivered in conjunction with other forms of interventions to enable self-management is a widely used intervention for ALWHIV on ART and is generally effective. Skills training with phone counseling interventions were applied in three studies and was effective [25, 32, 34]. Although the intervention period varied across these studies (Table 3), skills training with phone counseling interventions demonstrated effectiveness in improving many components of self-management. Peer-based HIV symptom management and skills training was another interventional approach to improve self-management, and in this case, significant improvement in the quality of life was reported [26]. This finding supported by three studies [31, 33, 35]. However, there was no change in adherence and symptom management with peer-based HIV symptom management programs and skills training. This suggests that peer-based interventions with skills training or other forms of intervention could be useful in improving some aspects of self-management but not others. Individualized skills training/educational sessions demonstrated significant improvement in many components of self-management other than CD4 count [27] and self-efficacy [30]. Overall, it appears that whilst approaches to skills training vary, they are generally effective, particularly in terms of improving psychosocial outcomes. Peer-based delivery of skills training appears less effective in improving physical and behavioral health outcomes.

The second most commonly applied intervention in the included studies was counseling (phone or general counseling) combined with different forms of interventions (for example skills training, symptom management manual, or education) with the intervention period lasting for 45 minutes to 90 minutes per session [25, 28, 30, 32, 34]. The counseling interventions were effective across all studies for most outcomes except self-efficacy [30] even though the mode of delivery varied across the included studies. Therefore, counseling with skill training, education, or symptom management manuals could be an essential intervention for improving the different aspects of self-management outcomes. However, self-efficacy for self-management remained unchanged in two studies [24, 30]. Improving self-efficacy for self-management may require more psychological and behavioral interventions with continuous phone counseling or online peer support.

Technology-assisted (via phone and website) interventions, in conjunction with other forms of interventions, were consistently found to be effective in improving self-management behaviors such as maintaining medication adherence, coping with HIV conditions and management of side effects [24]. Most of the interventions included in the review included phone calls as a means of follow-up or as a primary intervention to improve self-management and were effective [25, 26, 28, 29, 31, 34, 35]. All interventions that included phone calls for counseling were effective in improving many outcomes of self-management. The exception was quality of life and mobilizing social support, which were not significantly improved in one reported study [35]. Technology-assisted interventions such as phone calls and website- based interventions facilitate the sharing of information with ALWHIV efficiently and cost-effectively. The effectiveness of these types of intervention supported by the study conducted by Millard et al. [33]. In many parts of the world, ALWHIV may have access to cell phones, which allows information exchanges through calling, text messaging and the internet. Therefore, this type of intervention allows for remote access and timely information exchange. It also facilitates the connection between health providers, among peers and patients in resource-limited settings, by minimizing the cost of travel, saving time, and allowing privacy. These benefits may encourage the use of technology for improving self-management behaviors.

Interventions mainly targeting social support for improving self-management were effective in strengthening social relationships and social participation concerning their HIV conditions [24, 33]. However, Webel et al. [35] found no improvement in social support mobilization for self-management among the intervention group. The variability of effectiveness may be related to the mode of delivery. It could also be due to the common practice of searching for information from technology sources or the internet rather than seeking social support for self-managing HIV conditions. Although there were relatively few included studies that assessed social support, based on the current findings, it is possible to infer that social support may improve self-management.

A previous systematic review on a specific self-management education program [13], found that interventions focused on coping and symptom management improved the engagement of people living with HIV in self-management programs and the current review supports this finding. Interventions to improve self-management behaviors that focused on symptom management guidelines or coping behaviors were more effective than usual care [25–27, 34]. However, in two studies [26, 27], the intervention did not show a direct improvement in symptom management, although there were changes in quality of life and CD4 count. The possible explanation for this finding could be differences in the mode of delivery, duration, and type of intervention.

Whilst interventions designed to improve the self-management outcomes were generally found to be more effective than usual care [25, 27, 29, 31, 33–35], there was evidence of variability of effectiveness that could be related to a mix of contextual factors, intervention design features and implementation of the interventions. As the theory indicates [17], there is a complex inter-relationship between contextual factors and the process of self-management that influences a range of proximal and distal outcomes. Few studies provide a sufficiently detailed description of the intervention and its implementation in context to examine these relationships in detail. In particular, there is a lack of contextual grounding of the interventions, for example, in terms of the physical and social environment of study participants, or specific individual and family characteristics. The complex nature of self-management suggests that a multi-faceted, context-sensitive approach to intervention is required and there is unlikely to be a ‘one-size-fits-all’ solution. Equally, interventions designed to improve self-management need to be accessible and straightforward for users, particularly in resource-constrained settings.

### Limitations

The review has some limitations. The search did not include grey literature, with the potential that some relevant studies may have been missed. It was not possible to generate a single estimated effect size due to the heterogeneity of interventions and outcomes evaluated in different studies. Behind a simple description of an intervention such as ‘skills training’, there was a considerable variation related to the length and frequency of the intervention, how it was delivered, and by whom.

Furthermore, many of the studies involved multi-component interventions, for example, skills training plus counseling. This combination of the complexity of the interventions studied, and inconsistencies with outcome measurement made narrative synthesis and interpretation of the evidence challenging. The review attempted to deal with the complexity and resulting limitations by presenting evidence from both the intervention and outcomes perspective and providing summary tables of individual studies. Overall, many of the interventions studied produced positive benefits; however, it is not possible to make definitive recommendations regarding the most evidence-based way of designing self-management interventions for ALWHIV on ART in terms of which are the best interventions to combine, how to deliver the interventions and over what time. The lack of economic analyses within the included studies is an additional limitation in this regard. Another potential limitation of the review is the fact that the included studies were from high-income countries, mostly North America. No studies were included from Africa or other low and middle-income countries. This may affect the generalizability and applicability of the review to these countries where HIV prevalence is the highest.

## Conclusions

This review has synthesized evidence on interventions to improve the self-management of ALWHIV on ART. The evidence indicates that interventions that include components of skills training, counseling, symptom management instructions or manuals, technology-assisted teaching, and social support interventions were generally effective in improving physical, behavioral, and psychosocial self-management outcomes. Different forms of training combined with other modalities of interventions found to be effective in improving self-management outcomes, especially adherence to medication, quality of life, and symptom management. However, peer-based interventions seem less effective in improving different components of self-management. Further studies are required to give more attention to study design, description of interventions, and cost-effectiveness.

## Recommendations for practice

Based on the evidence in this review, health professionals are recommended to offer self-management skills training and counseling programs to ALWHIV on ART. There could be a benefit in providing resources such as a symptom management manual and encouraging the use of technology, for example, cell phone messaging and internet applications to enable fast and easy access to information.

## Supporting information

S1 Checklist PRISMA checklists filled. (DOC)

S1 Table Summary of evidence findings. (DOCX)

S1 Appendix Search strategy. (DOCX)

S2 Appendix Data extraction instrument. (DOCX)

S1 File (DOCX)
