# Supplementary material for: Interventions to improve self-management of adults living with HIV on Antiretroviral Therapy: A systematic review
Source: PLoS One. 2020 May 11;15(5):e0232709. doi: 10.1371/journal.pone.0232709 (PMC7213740; doi:10.1371/journal.pone.0232709)
Supplement: S1 Table — (DOCX) [file pone.0232709.s002.docx]

**Table 6: Summary of evidence findings**

| **Interventions to improve self-management include training, counselling, peer-mentoring, social support, providing self-management manuals, and encouraging active self-management compared to usual/standard care** | | | | | | |
| --- | --- | --- | --- | --- | --- | --- |
| **Patient or population**: Adults living with HIV on ART.  **Intervention**: interventions to improve self-management include training and or counselling and or peer-mentoring and or providing social support and or providing self-management manuals, and or encouraging active self-management  **Comparison**: usual/standard care. | | | | | | |
| Outcomes | **Anticipated absolute effects^*^** (95% CI) | | Relative effect (95% CI) | № of participants  (studies) | Certainty of the evidence (GRADE) | |
|  | **Risk with usual care** | **Risk with interventions** |  |  |  |  |
| Self-efficacy | not estimable | not estimable | not estimable | 394 (4 RCTs) | ⨁⨁⨁◯ MODERATE ^a^ |  |
| Social support mobilization | not estimable | not estimable | not estimable | 490 (3 RCTs) | ⨁⨁◯◯ LOW ^b, c, d^ |  |
| Adherence to ART | not estimable | not estimable | not estimable | 1151 (7 RCTs) | ⨁⨁⨁◯ MODERATE ^b, c, d^ |  |
| Symptom management | not estimable | not estimable | not estimable | 335 (3 RCTs) | ⨁⨁◯◯ LOW ^a, b, c, d^ |  |
| Quality of life | not estimable | not estimable | not estimable | 1143 (8 RCTs) | ⨁⨁⨁◯ MODERATE ^a, b, c, d^ |  |
| Coping | not estimable | not estimable | not estimable | 468 (3 RCTs) | ⨁⨁⨁◯ MODERATE ^a, b, c, d^ |  |
| ***The risk in the intervention group** (and its 95% confidence interval) is based on the assumed risk in the comparison group and the **relative effect** of the intervention (and its 95% CI).   **CI:** Confidence interval | | | | | | |
| **GRADE Working Group grades of evidence** **High certainty:** We are very confident that the true effect lies close to that of the estimate of the effect **Moderate certainty:** We are moderately confident in the effect estimate: The true effect is likely to be close to the estimate of the effect, but there is a possibility that it is substantially different **Low certainty:** Our confidence in the effect estimate is limited: The true effect may be substantially different from the estimate of the effect **Very low certainty:** We have very little confidence in the effect estimate: The true effect is likely to be substantially different from the estimate of effect | | | | | | |

**Explanations:** ^a^ Small sample size^, b^ Inconsistency in methods, ^c^ Difference in participants, ^d^ Different in tool
