# Supplementary material for: Interventions to improve self-management of adults living with HIV on Antiretroviral Therapy: A systematic review
Source: PLoS One. 2020 May 11;15(5):e0232709. doi: 10.1371/journal.pone.0232709 (PMC7213740; doi:10.1371/journal.pone.0232709)
Supplement: S1 Appendix — (DOCX) [file pone.0232709.s003.docx]

**Appendix I: Search strategy**

**Database(s): Pub Med from 2001 to March 30, 2019 with daily updates**

| Number | Searches | Items found |
| --- | --- | --- |
| #6 | Search [[1 AND #2 AND #3](#_ENREF_36)]; Full text; Publication date | **205** |
| #5 | Search [[1 AND #2 AND #3](#_ENREF_36)] Filters: Full text | 210 |
| #4 | Search [[1 AND #2 AND #3](#_ENREF_36)] | 216 |
| #3 | Search (Interventions[mh] OR Strategies[mh] OR Intervention[sh] OR Interventions*[tiab]) | 37,966 |
| #2 | Search (Self-management[mh] OR Self-management*[tiab] OR self-care[mh] OR self-care*[tiab]) | 66,656 |
| #1 | Search (HIV [mh] OR HIV [tiab] OR HIV-1*[tiab] OR HIV-2*[tiab] OR Human Immunodeficiency Virus*[tiab] OR AIDS virus*[tiab] OR Acquired Immune Deficiency Syndrome Virus*[tiab] OR Acquired Immunodeficiency Syndrome Virus*[tiab]) | 313,514 |

**Database(s): CINAHL from 2001 to March 30, 2019 with daily updates**

| Number | Searches | Items found |
| --- | --- | --- |
| S3 | ( MH Human Immunodeficiency Virus OR TI Human Immunodeficiency Virus* OR AB Human Immunodeficiency Virus* TI HIV OR AB HIV OR TI “Human Immunodeficiency Virus” OR AB “Human Immunodeficiency Virus” ) AND ( MH self-management OR TI self-management OR AB self-management OR TI “ self-care” OR AB “self-care” ) AND ( MW Interventions* OR TI Interventions* OR AB Interventions* )  Full Text; Abstract Available; Published Date, Narrow by Language: - English | **32** |
| S2 | ( MH Human Immunodeficiency Virus OR TI Human Immunodeficiency Virus* OR AB Human Immunodeficiency Virus* TI HIV OR AB HIV OR TI “Human Immunodeficiency Virus” OR AB “Human Immunodeficiency Virus” ) AND ( MH self-management OR TI self-management OR AB self-management OR TI “ self-care” OR AB “self-care” ) AND ( MW Interventions* OR TI Interventions* OR AB Interventions* )  Published Date and English | 101 |
| S1 | ( MH Human Immunodeficiency Virus OR TI Human Immunodeficiency Virus* OR AB Human Immunodeficiency Virus* TI HIV OR AB HIV OR TI “Human Immunodeficiency Virus” OR AB “Human Immunodeficiency Virus” ) AND ( MH self-management OR TI self-management OR AB self-management OR TI “ self-care” OR AB “self-care” ) AND ( MW Interventions* OR TI Interventions* OR AB Interventions* ) | 104 |

**Database(s): Embase from 2001 to March 30, 2019 with daily updates**

| Number | Searches | Item found |
| --- | --- | --- |
| #5 | #1 AND #2 AND #3 AND #4 | **86** |
| #4 | #3 AND ('clinical trial'/de OR 'controlled study'/de OR 'human'/de OR 'randomized controlled trial'/de) AND 'human immunodeficiency virus infection'/de AND (2000:py OR 2001:py OR 2002:py OR 2003:py OR 2004:py OR 2005:py OR 2006:py OR 2007:py OR 2008:py OR 2009:py OR 2010:py OR 2011:py OR 2012:py OR 2013:py OR 2014:py OR 2015:py OR 2016:py OR 2017:py OR 2018:py OR 2019:py) AND ('article'/it OR 'conference abstract'/it) AND ([adult]/lim OR [aged]/lim OR [middle aged]/lim OR [young adult]/lim) | 5,406 |
| #3 | ('mw interventions' OR ti) AND 'interventions' OR 'intervention':ti,ab | 748,281 |
| #2 | 'self-care'/de OR 'self-management':ti,ab OR 'self-care*':ti,ab OR 'selfcare':ti,ab | 64,103 |
| #1 | 'human immunodeficiency virus'/exp OR hiv:ti,ab OR 'hiv 1':ti,ab OR 'hiv 2':ti,ab OR 'human immunodeficiency virus':ti,ab OR 'aids virus':ti,ab OR 'acquired immune deficiency syndrome virus':ti,ab OR 'acquired immunodeficiency syndrome virus':ti,ab | 408,109 |

**Search from other sources found**: **10 articles**
