# Supplementary material for: Interventions to improve self-management of adults living with HIV on Antiretroviral Therapy: A systematic review
Source: PLoS One. 2020 May 11;15(5):e0232709. doi: 10.1371/journal.pone.0232709 (PMC7213740; doi:10.1371/journal.pone.0232709)
Supplement: S2 Appendix — (DOCX) [file pone.0232709.s004.docx]

**Appendix II: Critical Appraisal Checklist**
**JBI Critical Appraisal Checklist for Quasi-Experimental Studies (non-randomized experimental studies)**

Reviewer Date

Author Year Record Number

|  | Yes | No | Unclear | Not applicable |
| --- | --- | --- | --- | --- |
| 1. Is it clear in the study what is the “cause’ and what is the ‘effect’ (i.e. there is no confusion about which variable comes first)? | □ | □ | □ | □ |
| 1. Were the participants included in any comparisons similar? | □ | □ | □ | □ |
| 1. Were the participants included in any comparisons receiving similar treatment/care, other than the exposure or intervention of interest? | □ | □ | □ | □ |
| 1. Was there a control group? | □ | □ | □ | □ |
| 1. Were there multiple measurements of the outcome both pre and post the intervention/exposure? | □ | □ | □ | □ |
| 1. Was follow up complete and if not, were differences between groups in terms of their follow up adequately described and analyzed? | □ | □ | □ | □ |
| 1. Were the outcomes of participants included in any comparisons measured in the same way? | □ | □ | □ | □ |
| 1. Were outcomes measured in a reliable way? | □ | □ | □ | □ |
| 1. Was appropriate statistical analysis used? | □ | □ | □ | □ |

Overall appraisal: Include □ Exclude □ Seek further info □

Comments (Including reason for exclusion)

**JBI Critical Appraisal Checklist for Randomized Controlled Trials**

Reviewer Date

Author Year Record Number

|  | Yes | No | Unclear | NA |
| --- | --- | --- | --- | --- |
| 1. Was true randomization used for assignment of participants to treatment groups? | □ | □ | □ | □ |
| 1. Was allocation to treatment groups concealed? | □ | □ | □ | □ |
| 1. Were treatment groups similar at the baseline? | □ | □ | □ | □ |
| 1. Were participants blind to treatment assignment? | □ | □ | □ | □ |
| 1. Were those delivering treatment blind to treatment assignment? | □ | □ | □ | □ |
| 1. Were outcomes assessors blind to treatment assignment? | □ | □ | □ | □ |
| 1. Were treatment groups treated identically other than the intervention of interest? | □ | □ | □ | □ |
| 1. Was follow up complete and if not, were differences between groups in terms of their follow up adequately described and analyzed? | □ | □ | □ | □ |
| 1. Were participants analyzed in the groups to which they were randomized? | □ | □ | □ | □ |
| 1. Were outcomes measured in the same way for treatment groups? | □ | □ | □ | □ |
| 1. Were outcomes measured in a reliable way? | □ | □ | □ | □ |
| 1. Was appropriate statistical analysis used? | □ | □ | □ | □ |
| 1. Was the trial design appropriate, and any deviations from the standard RCT design (individual randomization, parallel groups) accounted for in the conduct and analysis of the trial? | □ | □ | □ | □ |

Overall appraisal: Include □ Exclude □ Seek further info □

Comments (Including reason for exclusion)
