# Supplementary material for: Interventions to improve self-management of adults living with HIV on Antiretroviral Therapy: A systematic review
Source: PLoS One. 2020 May 11;15(5):e0232709. doi: 10.1371/journal.pone.0232709 (PMC7213740; doi:10.1371/journal.pone.0232709)
Supplement: S1 File — (DOCX) [file pone.0232709.s005.docx]

GRADEpro GDT: GRADEpro Guideline Development Tool [Software]. McMaster University, 2015 (developed by Evidence Prime, Inc.). Available from [gradepro.org](https://gradepro.org/cite/gradepro.org).
